# The music of trees: the intergenerative tie between primary care and public health

**DOI:** 10.1080/17571472.2016.1152100

**Published:** 2016-03-09

**Authors:** Peter Whitehouse

**Affiliations:** ^a^ Department of Neurology, Case Western Reserve University, Cleveland, OH, USA; ^b^ University of Toronto, Toronto, ON, Canada; ^c^ Intergenerational Schools International, Cleveland, OH, USA

**Keywords:** Metaphor, narrative, ecology, public health, arts

## Abstract

Stories help us frame and understand complex ideas and challenges. Metaphors are particularly powerful linguistic devices that guide and extend our thinking by bridging conceptual domains, for example to consider the brain as a digital computer. Trees are widely used as metaphors for broad concepts like evolution, history, society, and even life itself, i.e. ‘the tree of life’. Tree-like diagrams of roots and branches are used to demonstrate historical and cultural relationships, for example, between different species or different languages. In this paper, we describe a theatrical character called a tree doctor which is a living metaphor. A human being, namely the author, lectures, acts or dances as a tree and offers lessons to Homo Sapiens about ‘holistic’ ideas of health. The character teaches us to not only see the value of our relationships to trees, but the importance of seeing forests as well the individual trees. The metaphorical statement that we should not ‘miss the forest for the trees’ means we should learn to think of health embedded in systems and communities. In medicine, we too often focus on individual molecules, pharmaceuticals, or even patients and miss the bigger picture of public and environmental health. In a time of great ecological system change, the tree doctor points to broad ethical responsibility for each other and future generations of humans and other living creatures. The character embraces arts and particularly music as a powerful way of infusing purpose and improving the qualities of our lives together, especially as we age. The tree doctor knows the value of intergenerational relationships. But it also points to intergenerative innovations across many cultural domains, disciplines and professions. The tree doctor supports primary care and empowers the value of intergenerational relationships, art and music in the recommendations doctors make to patients to improve their health and well-being.

## Why this matters to me

Let me explain (or try to). I am a geriatric neurologist, cognitive neuroscientist, psycholinguist, environmental bioethicist, nature photographer and a very new, first time grandfather who has always been a primary care wannabe, admiring the comprehensive, long-term nature of that physician–patient relationship. I trained at Johns Hopkins as an MD–PhD (psychology) and neurologist with further training in psychiatry and neuroscience. I became a specialist, gaining fame and fortune at least for a fairly brief moment in time for being the world’s expert on a single brain nucleus in dementia (the ironically originally named substantia innominata or now cholinergic basal forebrain). Now I work in environmental and population health, focusing on ethics, school-based health care, and ageing. My latest career move as a healer was to invent a performance character, a metaphorical tree doctor. When the new me first appeared in public in 2013, it was in an arboretum outside of Toronto, home of the Legacy Project (www.legacyproject.org), as part of an intergenerational international environmental sustainability event. I was asked by a fellow participant whether I was the kind of tree doctor that cut-off damaged tree limbs. I corrected the questioner with an element of humour by saying that was actually performed by a tree surgeon, but in actual fact I meant something quite different, i.e. that I was a tree doctoring human beings, not the other way around. I also pretty much invented the word ‘intergenerative’.

### Key messages

Stories and especially metaphors matter in the health of individuals and communities

Performance art can contribute to opening minds and hearts

Bioethics needs to expand its moral scope

The human species is in life-threatening cultural and ecological trouble

Primary care is primed to adopt new ways of communicating about health

**Ethical Review** not needed for personal opinion piece

Metaphors are narrative and cognitive devices that bridge between (‘meta’) mental carrying vessels (‘phores’). They are powerful conceptual processes for transferring and blending ideas from often disconnected corners of our minds to another and sharing novel ideas throughout communities of thought. They are also relevant to health and primary care.

In the past, natural philosophers and in modern times neuroscientists have used all kinds of metaphors to guide both our theories of neural function and practices in trying to understand the brain. The ancients explored the idea that brains are like radiators to cool the blood. Getting closer to our times, brains became machines, compared to hydraulic pumps and then electric generators. Finally in modern times, the dominant metaphor for the brain became the digital computer. What does it mean to think of the carbon-based, organic brain as a silicon-based, digital information processing device? What are the similarities and differences? What can we learn from one about the other? Metaphors are intergenerative –meaning innovating through integration or blending and going ‘between’ to go ‘beyond’.

## Climbing the heights of metaphor

Trees are ultimate metaphors – in fact they could be considered megametaphors. They are commonly used metaphors for very big ideas like biological evolution, history, culture, and even for life itself. Numerous traditions celebrate the idea of a ‘tree of life’. And in the brain, the tree is a metaphor for neurons themselves as they interact through their dendritic ‘arborizations’. We all use banks to deposit money but I shifted from being a brain banker to a story banker.[1] No wonder that my own journey as a healer from neuromolecules to mental maps and memories to narrative medicine led me to metaphorical trees.

What can individual human beings and communities learn from trees about staying healthy? Trees connect earth and sky. They are also strongly associated with other fundamental elements like water and fire. Trees celebrate the importance of roots, understanding from where you came. They reach for the sky and the sun, aspirationally through their leaves. Scientifically, they exhibit geotropism orienting to gravity, and phototropism towards light. They teach us about time, not only with gorgeous cyclic, often colourful lessons of the passing of the seasons in a single year and rings of growth that record the events of each year, but with lifespans that can far exceed those of human beings. My tree doctor character was born slowly in the early years of the twenty-first century in the Muir Woods outside of San Francisco surrounded by redwoods hundreds of feet tall and thousands of years old. I visit that area of Marin County quite often as I worked with Rachel Remen’s Healer’s Art programme and other activities at Commonweal (www.commonweal.org).

## Even trees can fall

Yet even these giant trees are threatened by human activity whether it be by those who damage them by physical contact, or coal plants that create global climate change, which in turn disrupts weather patterns, such as diminishing the fogs of San Francisco Bay from which these trees gain critical moisture. As sheltering umbrellas, trees teach us about the relationships between living creatures in a single ecological community and even the protectiveness of mature trees for growing seedlings. Trees serve as shelter for all kinds of other creatures in their circle of influence. Forests protect entire species of arboreal creatures. And trees demonstrate the wonder of birth and development, as, for example, acorns becoming mature trees and about death, as in life that emerges from a fallen tree trunk. They teach us about the value of interdependence, not just independence. The hundreds of wildfires affecting the United States and other countries teaches us that fire, water, air and earth are in dynamic balance and all are necessary. But we human beings in our foolishness and for our own short-term material purposes have disrupted life rhythms in the forests about which we understand so little.

## From the jungle to public health

Trees as metaphors teach us to see the ‘forest as well as the trees’ i.e. seeing the big picture from a systems perspective as well as individual component parts. Individual trees, just as individual human beings, are magnificent, but both living creatures need to be in community in order to stay healthy. My tree character matured in the Brazilian Amazon forest in 2011. While reading about the power of narrative in shamanism on the plane ride down, I came to appreciate scientifically and spirituality the artificial way in which modern medical science separates individual and population health, something a shaman would not do. From that moment on, I became a policy advocate for bridging primary care and public health. It also inspired me to continue the difficult work of creating school-based health care where individual and community health should be blended, just as the educational curriculum should integrate with the health care system.[2] Trees teach us about systems thinking and deeper bioethics.

## Trees as artful and ethical

Metaphor and trees have also led me to intensify my work in bridging art and science in health. For example, I am currently leading an effort at the American Public Health Association to develop a policy through the Ethics Section on the importance of art and the humanities for public health. The original conceptions of bioethics incorporated environmental ideas and the humanities in their concepts but modern mainstream bioethics has become infused with abstract theory (‘principles’) and medicine-mimicking empiricism. Rather than serving as a critic of biomedicine, bioethics has too often become co-opted by the agenda (and money) of medicine. The art of clinical medicine (and science) is often lost in the quest for ever more expensive biomarkers for an ever-growing pharmaceutical industry-driven list of diseases. I have been advocating for a deeper bioethics that does not separate clinical, public health and environmental ethics and blends in a strong aesthetic element.[3]

The critical thought-provoking nature of the humanities, not to mention their humanising nature is subverted by scientism as we seek to cure every form of human suffering, including brain ageing itself (formerly called the various forms of Alzheimer’s disease). Science has lost its aesthetic for seeking healthy holism by reducing humans to molecular genetic forms through evangelical genomics. Art might once again help our species correct our presumptions that capitalistic-driven, technoscience solutions are the answer to all human challenges. Perhaps it is not too late to recognise that national ‘development’ means more that economics and individual ‘wealth’ more than money. Certainly trees rise above such narrow human conceptions of life and yet fall prey to being commodified themselves.

Trees make music. Have you ever heard and watched the music and dance of a forest with the wind? Music plays a key role as a form of art. It seems to dwell in special albeit distributed places in the brain and possess special healing properties. The music of the leaves and branches of individual trees is awesome but in the company of others the symphony of forest music is awe-inspiring. As a tree doctor, I love music personally and recommended it professionally. Dancing adds the dimension of physical movement. So what is special about music? The recent death of one of my neurological narrative heroes Oliver Sacks reminds us of the power of both music and narrative. We are all musicophilic one way or another – cultural variations in genre might mask the basic fundamental nature of rhythm. As a review in this current issue shows there is much evidence to support the value of music especially as we age. ‘Music of life’ shows how a variety of different programmes, especially when conducted socially, can improve well-being and quality of life.[4] In their review of the literature Hallam and Creech find:

Participation in a wide range of musical activities has been shown to provide a source of enhanced social cohesion, enjoyment, personal development and empowerment for older people supporting collaborative learning, friendship, a sense of belonging, enhanced subjective wellbeing, and access to new social roles and relationships. Music can also provide a sense of contentment, satisfaction and peace.

Their work reaffirms the value of intergenerational programming:

Some of the older people from the sheltered housing community engaged in intergenerational activities with children from local primary schools. This energized them, was enjoyable and gave them an opportunity to relate to the younger generation. The intergenerational activity was an opportunity for different generations to socialize, show respect for each other and enjoy each other’s company.

Yet the Cochrane Collaboration review of music therapy [5] gives lukewarm support for its value, at least with people with dementia. Is that the problem of music therapy or of the nature of evidence? Music therapy focuses mainly on interventions for individuals or small groups. Music is often a community event and might best be viewed as a public health intervention to improve community, as well as individual, health and well-being. As mentioned above, a policy statement for the APHA on the value of art and humanities for public health is being drafted through the Ethics Section. In that process, we will ask what constitutes evidence to move policy forward. Anecdotes are devalued in medicine and the randomised controlled trial reigns epistemologically supreme. Yet arguably, stories are more powerful than data in our private and public lives. And the weakness of RCTs, like narrow scope and poor generalisability, and their distorted designs and interpretation, particularly by industry, not often realised. A creative intergenerative blend of qualitative and quantitative mixed methods is needed. And we must remember that the most important things in life are the hardest to measure. Who thinks that measuring height, girth and age of a tree captures what is most essential about trees?

## Trees provide big data

The editor wanted data in this paper. Hence, I thought I should include some information about trees in relation to humans. There are estimated to be three trillion trees in the world, 400 for every human being. More trees die every year than that are born, leaving a net deficit of 5 billion a year; the main causes of net loss are human agriculture plus industry and urban sprawl.[6] Trees are air breathers - the lungs of the planet. Humans are complementary air breathers as oxygen and carbon dioxide flow in and out of our respiratory mechanisms in opposite directions to that of trees. Both species depend on water and participate in the slow processes of evolution. Yet we can imagine that we are a tree and to my knowledge no actual tree has thought of themselves as being sentient. Apparently it remains to our species to demonstrate that having advanced nervous systems that miraculously led to something we call consciousness actually provides evolutionary advantage in the long term. At the moment, this tree doctor is not at all sure we will accomplish this task of demonstrating the evolutionary value of self and other awareness. Despite our amazing individual and collective memories and our abilities to imagine various alternative realties and futures, we need to develop our sense of presence and celebrate the moment, ideally together in purposeful solidarity with life.

## Back to the roots of primary care

And coming back to primary care and my wannabe status, I can only say that the tree doctor has taught me that care is primary and primary care should be the prime investment in health. And art and music are primary too and worthy of further investment. Recommend your patients to listen deeply to the stories inside themselves and about in the world. Know where the nature centres and arts and music programmes are near your practice. Advocate for broad public health approaches. Live your life resonant with nature and give more than a thought for the future. Until we appreciate the journey from life to death is not one full of easy answers and that cures are not just around the next well-funded scientific corner, patients will not come to appreciate, as this tree doctor does, the awe-inspiring devotion and skill of the primary care doctor, nurse or other healers.

**Governance** Case Western Reserve University and University of Toronto

## Disclosure statement

I occasionally perform but am rarely paid as a tree doctor.
